# Foliar Growth Regulator Sprays Induced Tolerance to Combined Heat Stress by Enhancing Physiological and Biochemical Responses in Rice

**DOI:** 10.3389/fpls.2021.702892

**Published:** 2021-07-23

**Authors:** Alvaro Daniel Pantoja-Benavides, Gabriel Garces-Varon, Hermann Restrepo-Díaz

**Affiliations:** ^1^Universidad Nacional de Colombia, Sede Bogotá, Facultad de Ciencias Agrarias, Departamento de Agronomía, Bogotá, Colombia; ^2^Federación Nacional de Arroceros, Seccional Saldaña, Saldaña, Colombia

**Keywords:** stomatal conductance, Fv/Fm ratio, high daytime and nighttime temperature, lipid peroxidation, plant acclimatization

## Abstract

Rice yield has decreased due to climate variability and change in Colombia. Plant growth regulators have been used as a strategy to mitigate heat stress in different crops. Therefore, this study aimed to evaluate the effect of foliar applications of four growth regulators [auxins (AUX), brassinosteroids (BR), cytokinins (CK), or gibberellins (GA)] on physiological (stomatal conductance, total chlorophyll content, F_v_/F_m_ ratio, plant canopy temperature, and relative water content) and biochemical (Malondialdehyde (MDA) and proline contents) variables in two commercial rice genotypes exposed to combined heat stress (high day and nighttime temperatures). Two separate experiments were carried out using plants of two rice genotypes, Fedearroz 67 (“F67”) and Fedearroz 2000 (“F2000”) for the first and second experiments, respectively. Both trials were analyzed together as a series of experiments. The established treatments were as follows: absolute control (AC) (rice plants grown under optimal temperatures (30/25°C day/nighttime temperatures), heat stress control (SC) [rice plants only exposed to combined heat stress (40/30°C)], and stressed rice plants and sprayed twice (5 days before and after heat stress) with a plant growth regulator (stress+AUX, stress+BR, stress+CK, or stress+GA). The results showed that foliar CK sprays enhanced the total chlorophyll content in both cultivars (3.25 and 3.65 mg g^−1^ fresh weight for “F67” and “F2000” rice plants, respectively) compared to SC plants (2.36 and 2.56 mg g^−1^ fresh weight for “F67,” and “F2000” rice plants, respectively). Foliar CK application also improved stomatal conductance mainly in “F2000” rice plants compared to their heat stress control (499.25 vs.150.60 mmol m^−2^s^−1^). Foliar BR or CK sprays reduced plant canopy temperature between 2 and 3°C and MDA content in plants under heat stress. The relative tolerance index suggested that foliar CK (97.69%), and BR (60.73%) applications helped to mitigate combined heat stress mainly in “F2000” rice plants. In conclusion, foliar BR or CK applications can be considered an agronomic strategy to help to ameliorate the negative effect of combined heat stress conditions on the physiological behavior of rice plants.

## Introduction

Rice (*Oryza sativa*) belongs to the Gramineae family and is one of the most cultivated cereals around the world together with maize and wheat (Bajaj and Mohanty, 2005). Rice occupied 617,934 ha with a national production volume of 2,937,840 t and an average yield of 5.02 t ha^−1^ in 2020 (Federarroz (Federación Nacional de Arroceros), 2021).

Rice crops are being affected by global warming, causing different types of abiotic stress, such as high temperatures and drought periods (Wassmann et al., 2009; Chandio et al., 2020). Climate change has increased the global temperature around the world; it has been predicted that temperature will increase by 1.0–3.7°C during the twenty-first century, which will potentially increment the frequency and magnitude of heat stress events (IPCC, 2013). This increase in environmental temperatures has affected rice, generating a decrease between 6 and 7% in crop production (Lesk et al., 2016). On the other hand, climate variability has also generated adverse environmental conditions in crops such as periods of intense drought or high temperatures in the tropical and subtropical regions (Feller and Vaseva, 2014). Additionally, variability phenomena such as the “El Niño” phenomenon can cause heat stress conditions and aggravate the damage on crops in some tropical regions (Iizumi et al., 2014; Amanullah et al., 2017). In Colombia, rice-producing areas will expect increases in temperatures between 2 and 2.5°C by 2050, reducing rice yields and making an impact on product flows to markets and supply chains (Ramirez-Villegas et al., 2012).

The majority of rice crops are grown in regions where temperatures are close to the optimum range for crop growth (Shah et al., 2011). It has been reported that the optimal average temperatures for rice growth and development are usually 28 and 22°C for day and night, respectively (Kilasi et al., 2018; Calderón-Páez et al., 2021). Temperatures above those thresholds can cause moderate or severe heat stress periods during sensitive rice development stages (tillering, anthesis, flowering, and grain filling), negatively affecting grain yield (Shah et al., 2011). These yield reductions have been mainly associated with prolonged heat stress periods (more than 7 days) that affect plant physiology (Shah et al., 2011; Sánchez-Reinoso et al., 2014). Heat stress can cause a series of irreversible damages in plant metabolism and development due to the interaction of different factors such as the stress duration, or the maximum temperature reached (Porch Clay and Hall, 2013; Zhou et al., 2014).

Heat stress affects different physiological and biochemical processes in plants (Bita and Gerats, 2013; Chavez-Arias et al., 2018). Leaf photosynthesis is one of the most susceptible processes to heat stress in rice plants since the photosynthetic rate can decrease by 50% when daytime temperatures exceed 35°C (Restrepo-Diaz and Garces-Varon, 2013). The physiological response varies according to the type of heat stress in rice plants. For example, the photosynthetic rate and stomatal conductance are inhibited when plants are exposed to high day temperatures (33–40°C) or to a combination of high day and night temperatures (35 to 40°C for day, and 28 to 30°C for night) (Lü et al., 2013; Fahad et al., 2016; Chaturvedi et al., 2017). A high nighttime temperature (30°C) caused a moderate inhibition of photosynthesis but enhanced nighttime respiration (Fahad et al., 2016; Alvarado-Sanabria et al., 2017). Heat stress also affects leaf chlorophyll content, the ratio of variable to maximum chlorophyll fluorescence (F_v_/F_m_), and the activation of Rubisco in rice plants regardless of the period of stress (Cao et al., 2009; Yin et al., 2010; Sánchez-Reinoso et al., 2014).

Biochemical changes are another aspect of plant acclimatization to heat stress (Wahid et al., 2007). Proline content has been used as a biochemical indicator of stress in plants (Ahmed and Hassan, 2011). Proline plays an important role in plant metabolism since it acts as a source of C or N and as a membrane stabilizer under high temperature conditions (Sánchez-Reinoso et al., 2014). High temperatures also affect membrane stability by lipid peroxidation, leading to malondialdehyde (MDA) production (Wahid et al., 2007). Therefore, the MDA content has also been used to know the structural integrity of cell membranes under heat stress (Cao et al., 2009; Chavez-Arias et al., 2018). Finally, combined heat stress [37/30°C (day/night)] increased the electrolyte leakage percentage and MDA content in rice plants (Liu et al., 2013).

The use of plant growth regulators (GRs) has been evaluated to mitigate the negative effects of heat stress since these substances are actively involved in plant responses or mechanisms to develop physiological protection against this kind of stress (Peleg and Blumwald, 2011; Yin et al., 2011; Ahammed et al., 2015). The exogenous application of GRs has shown a positive response in plant tolerance to heat stress in different crops. Studies have observed that plant hormones such as gibberellins (GA), cytokinins (CK), auxins (AUX), or brassinosteroids (BR) have caused an increase in different physiological and biochemical variables (Peleg and Blumwald, 2011; Yin et al., 2011; Mittler et al., 2012; Zhou et al., 2014). In Colombia, the exogenous application of GRs and their effects are still not well-elucidated or studied in rice crops. However, a previous study showed that foliar BR applications enhanced rice tolerance by improving leaf gas exchange properties, chlorophyll, or proline content in rice seedlings (Quintero-Calderón et al., 2021).

Cytokinins mediate plant responses to abiotic stress, including heat stress (Ha et al., 2012). Also, it has been reported that exogenous applications of CK can reduce heat damages. For example, exogenous applications of zeatin improved the photosynthesis rate, chlorophyll *a* and *b* contents, and electron transport efficiency during heat stress in creeping bentgrass (*Agrotis estolon*í*fera*) (Xu and Huang, 2009; Jespersen and Huang, 2015). Exogenous applications of zeatin also improved the antioxidant activity and enhanced the synthesis of different proteins, decreasing the damage of reactive oxygen species (ROS), and the production of malondialdehyde (MDA) in plant tissues (Chernyad'ev, 2009; Yang et al., 2016; Kumar et al., 2020).

Gibberellic acid applications have also exhibited a positive response to heat stress. Studies have shown that GA biosynthesis mediates different metabolic pathways and enhances tolerance under high temperature conditions (Alonso-Ramirez et al., 2009; Khan et al., 2020). Abd El-Naby et al. (2020) found that exogenous foliar application of GA (25 or 50 mg^*^L) increased the photosynthetic rate and antioxidant activity in heat-stressed orange plants compared to control plants. It has also been observed that the exogenous application of GA enhanced the relative water content, chlorophyll and carotenoids content, and diminished lipid peroxidation in date palm (*Phoenix dactylifera)* under heat stress (Khan et al., 2020). Auxins also play an essential role in regulating adaptive growth responses to high temperature conditions (Sun et al., 2012; Wang et al., 2016). This growth regulator works as a biochemical marker during different processes such as the synthesis or degradation of proline under abiotic stress (Ali et al., 2007). Also, AUX enhance the antioxidant activity, causing a decrease in MDA in the plant because of lower lipid peroxidation (Bielach et al., 2017). Sergiev et al. (2018) observed that the proline content increased when garden pea (*Pisum sativum*) plants were treated with auxin analogs [1-(2-chloroethoxycarbonyl –methyl)-4-naphthalene sulfonic acid calcium salt (TA-12) and 1-(2-dimethylaminoethoxicarbonylmethyl) naphthalene chlormethylate (TA-14)] under heat stress. In the same experiment, they also observed that the MDA levels were lower in treated plants compared to plants without AUX application.

Brassinosteroids are another group of growth regulators that have been used to mitigate the effects of heat stress. Ogweno et al. (2008) reported that exogenous BR sprays increased the net photosynthetic rate, stomatal conductance, and maximum carboxylation rate of Rubisco in tomato (*Solanum lycopersicum*) plants under heat stress for 8 days. The foliar spray of epibrassinosteroid caused a higher net photosynthetic rate in cucumber (*Cucumis sativus*) plants under heat stress (Yu et al., 2004). Also, exogenous applications of BR delayed chlorophyll degradation and enhanced water use efficiency and maximum quantum yield of PSII photochemistry in heat-stressed plants (Holá et al., 2010; Thussagunpanit et al., 2015).

Rice crops are facing periods of high day and night temperatures due to climate change and variability (Lesk et al., 2016; Garcés, 2020; Federarroz (Federación Nacional de Arroceros), 2021). In plant phenotyping, the use of plant nutrients or biostimulants has been studied as a strategy to mitigate heat stress in rice-cultivating areas (Alvarado-Sanabria et al., 2017; Calderón-Páez et al., 2021; Quintero-Calderón et al., 2021). Additionally, the use of biochemical and physiological variables (leaf canopy temperature, stomatal conductance, chlorophyll fluorescence parameters, chlorophyll and relative water contents, malondialdehyde and proline synthesis) are reliable tools to screen rice under heat stress at local and international levels (Sánchez-Reinoso et al., 2014; Alvarado-Sanabria et al., 2017; Sarsu et al., 2018). However, research on the use of foliar plant hormone sprays in rice plants is still scarce at the local level. Therefore, the study of physiological and biochemical responses to the use of plant growth regulators is important to propose practical agronomic strategies to ameliorate the negative effects of combined heat stress periods in rice. In consequence, the objective of this research was to evaluate the effect of the foliar applications of four plant growth regulators (AUX, CK, GA, and BR) on the physiological (stomatal conductance, chlorophyll fluorescence parameters, and relative water content) and biochemical (photosynthetic pigments, malondialdehyde, and proline content) variables of two commercial rice genotypes subjected to combined heat stress (high day/nighttime temperatures).

## Materials and Methods

### General Growth Conditions and Plant Material

Two separate experiments were carried out in this study. Rice seeds of genotypes Fedearroz 67 (F67: genotype bred under high-temperature conditions in the last decade) and Fedearroz 2000 (F2000: genotype bred in the last decade of the twentieth century that shows tolerance to White Leaf Virus) were used in the first and second experiments, respectively. Both genotypes are widely grown by Colombian farmers. Seeds were sown in 10 L trays (39.6 cm length × 28.8 cm width × 16.8 cm height), containing sandy loam soil with 2% organic matter. Five pregerminated seeds were planted in each tray. The trays were set in a greenhouse at the Faculty of Agricultural Sciences of the Universidad Nacional de Colombia, Bogotá campus (43°50′56″ N, 74°04′051″ W), at an altitude of 2,556 m above sea level (m.a.s.l.), from October to December 2019 for the first experiment (Fedearroz 67), and in the same season in 2020 for the second experiment (Fedearroz 2000).

The environmental conditions in the greenhouse during each sowing season were as follows: 30/25°C day/night temperature, 60–80% relative humidity, and a natural photoperiod of 12 h (photosynthetically active radiation of 1,500 μmol (photons) m^−2^ s^−1^ at noon). At 20 days after seed emergence (DAE), plants were fertilized following the amounts of each element according to Sánchez-Reinoso et al. (2019): 670 mg of N per plant, 110 mg of P per plant, 350 mg of K per plant, 68 mg of Ca per plant, 20 mg of Mg per plant, 20 mg of S per plant, 17 mg of Si per plant, 10 mg of B per plant, 17 mg of Cu per plant, and 44 mg of Zn per plant. Rice plants were maintained under those conditions up to 47 DAE in each experiment as rice plants reached the phenological stage V5 during that period. Previous studies have shown that this phenological stage is an appropriate time to perform heat stress studies in rice plants (Sánchez-Reinoso et al., 2014; Alvarado-Sanabria et al., 2017).

### Heat Stress and Plant Hormone Treatments

Two separated applications of foliar growth regulators were performed in each experiment. The first group of foliar plant hormone sprays was performed 5 days before heat stress treatments (42 DAE) to prepare plants for environmental stress. Then, the second foliar sprays were carried out 5 days after subjecting plants to stress conditions (52 DAE). Four plant hormones were used, and the characteristics of each active ingredient sprayed in this study are mentioned in Supplementary Table 1. The concentrations of the foliar growth regulators used were as follows: (i) auxins (1-Naphthaleneacetic acid: NAA) at 5 × 10^−5^ M; (ii) gibberellins (Gibberellic acid: GA3) at 5 × 10^−5^ M; (iii) cytokinins (Trans-Zeatin) at 1 × 10^−5^ M; and (iv) brassinosteroids [Spirostan-6-one, 3,5-dihydroxy-, (3b,5a,25R)] at 5 × 10^−5^ M. These concentrations were selected since they showed a positive response and increased plant tolerance to heat stress (Zahir et al., 2001; Wen et al., 2010; El-Bassiony et al., 2012; Salehifar et al., 2017). Rice plants without any plant growth regulator sprays were only treated with distilled water. All rice plants were sprayed using a hand sprayer. The application volume was 20 ml H_2_O per plant, wetting the upper and lower leaf surfaces. All foliar applications were performed with an agricultural adjuvant (Agrotin, Bayer CropScience, Colombia) at a dose of 0.1% (v/v). The distance between the pot and the atomizer sprayer was 30 cm.

Heat stress treatments were carried out 5 days after the first foliar sprays (47 DAE) in each experiment. Rice plants were transferred from the greenhouse to growth chambers of 294 L of capacity (MLR-351H, Sanyo, Illinois, US) to establish heat stress or continue with the previous environmental conditions (47 DAE). The combined heat stress treatment was performed by setting the chambers with the following day/nighttime temperatures: a period of high daytime [40°C for 5 h (from 11:00 to 16:00)] and nighttime [30°C for 5 h (from 19:00 to 24:00)] for 8 consecutive days. Stress temperatures and exposure time were selected based on previous studies (Sánchez-Reinoso et al., 2014; Alvarado-Sanabria et al., 2017). On the other hand, a group of plants transferred to the growth chamber was kept at the same temperature of the greenhouse (30°C day/25°C night) for 8 consecutive days.

At the end of the experiment, the following groups of treatments were obtained: (i) growth temperature conditions + distilled water applications [absolute control (AC)], (ii) heat stress conditions + distilled water applications [heat stress control (SC)], (iii) heat stress condition + auxins (AUX) applications, (iv) heat stress condition + gibberellins (GA) applications, (v) heat stress condition + cytokinins (CK) applications, and (vi) heat stress condition + brassinosteroids (BR) applications. These groups of treatments were used in both genotypes (F67 and F2000). All treatments were arranged in a completely randomized design with five replicates, and each replicate consisted of one plant. Each plant was used for readings of the variables determined at the end of the experiment. The experiment lasted 55 DAE.

### Stomatal Conductance and Relative Water Content (RWC)

Stomatal conductance (*g*_*s*_) was determined using a portable porometer (SC-1, METER Group Inc., USA) with a range from 0 to 1,000 mmol m^−2^s^−1^, and a sample chamber aperture of 6.35 mm. Measurements were taken by clipping the sensor of the porometer onto a fully expanded mature leaf from the main tiller of the plant. *g*_*s*_ readings were taken between 11:00 and 16:00 h on three leaves per plant in each treatment, and values were averaged.

The RWC was determined following the method described by Ghoulam et al. (2002). A fully expanded leaf used to determine *g*_*s*_ was also used to measure RWC. The fresh weight (FW) was immediately determined after harvesting using a digital balance. Subsequently, leaves were placed inside plastic recipients with water under dark conditions and room temperature (22°C) for 48 h. Then, they were weighed on a digital balance to record the turgid weight (TW). Turgid leaves were dried in an oven at 75°C for 48 h and their dry weight (DW) was registered.

### Relative Chlorophyll Content and Chlorophyll α Fluorescence Parameters

The relative chlorophyll content was determined using a chlorophyll meter (atLeaf meter, FT Green LLC, US) and expressed as atLeaf units (Dey et al., 2016). The readings of maximum quantum efficiency of PSII (F_v_/F_m_ ratio) were recorded using a continuous excitation chlorophyll fluorimeter (Handy PEA, Hansatech Instruments, UK). Before taking F_v_/F_m_ measurements, leaves were adapted to darkness using leaf clips for 20 min (Restrepo-Diaz and Garces-Varon, 2013). After leaf adaptation in the dark, Baseline (F_0_) and maximum fluorescence (F_m_) were measured. The variable fluorescence (F_v_ = F_m_ – F_0_), the ratio of variable to maximum fluorescence (F_v_/F_m_), the maximum quantum yield of PSII photochemistry (F_v_/F_0_), and the F_m_/F_0_ ratio were calculated based on these data (Baker, 2008; Li et al., 2017). The readings of relative chlorophyll and chlorophyll fluorescence parameters were taken on the same leaves used for *g*_*s*_ readings.

### Biochemical Variables: Leaf Photosynthetic Pigments, Lipid Peroxidation (Malondialdehyde-MDA), and Proline

Approximately 800 mg of leaf fresh weight was collected for biochemical variables. Then, leaf samples were homogenized in liquid nitrogen and stored for further analysis. The spectral determination method used to estimate chlorophyll *a, b*, and carotenoid content on tissue was based on the methodology and the equations described by Wellburn (1994). Leaf tissue samples (30 mg) were collected and homogenized in 3 mL of 80% acetone. Then, the samples were centrifuged (Model 420101, Becton Dickinson Primary Care Diagnostics, USA) at 5,000 rpm for 10 min to remove particles. The supernatant was diluted to a final volume of 6 ml by adding 80% acetone (Sims and Gamon, 2002). Chlorophyll content was determined at 663 (chlorophyll *a*) and 646 (chlorophyll *b*) nm, whereas carotenoids were estimated at 470 nm using a spectrophotometer (Spectronic BioMate 3 UV-vis, Thermo, USA).

The thiobarbituric acid (TBA) method described by Hodges et al. (1999) was used to estimate membrane lipid peroxidation (MDA). Approximately 0.3 g of leaf tissue was also homogenized in liquid nitrogen. The samples were centrifuged at 5,000 rpm, and then the absorbances were measured at 440, 532, and 600 nm with the spectrophotometer. Finally, an extinction coefficient (157 M·ml^−1^) was used to obtain the MDA concentration.

Proline content was determined for all treatments using the method described by Bates et al. (1973). Ten milliliters of a 3% aqueous solution of sulfosalicylic acid was added to the stored samples, which were then filtered with Whatman paper (No. 2). Subsequently, 2 mL of this filtrate was reacted with 2 mL of ninhydrin acid and 2 mL of glacial acetic acid. The mixture was placed in a water bath at 90°C for 1 h. The reaction was stopped by incubation on ice. The resulting solution was dissolved in 4 mL of toluene by shaking the test tubes vigorously using a vortex shaker. Absorbance readings were determined at 520 nm with the same spectrophotometer used in the quantification of photosynthetic pigments (Spectronic BioMate 3 UV-Vis, Thermo, Madison, WI, USA).

### Plant Canopy Temperature and Crop Stress Index (CSI)

The methodology described by Gerhards et al. (2016) was used to calculate plant canopy temperature and CSI. Thermal photographs were taken with a FLIR 2 camera (FLIR Systems Inc., Boston, MA, USA) with an accuracy of ±2°C at the end of the stress period. A white surface was placed behind plants for photography. Similarly, two plants were considered as the reference pattern. These plants were placed on a white surface; one of them was covered with an agricultural adjuvant (Agrotin, Bayer CropScience, Bogotá, Colombia) to simulate the opening of all stomata [wet pattern (Twet)], and the other was a leaf without any application [dry pattern (Tdry)] (Castro-Duque et al., 2020). The photography was taken with a distance of 1 m between the camera and the pot.

### Relative Tolerance Index (RTI)

The relative tolerance index was calculated indirectly to determine the tolerance of the treated genotypes evaluated in this research, using the stomatal conductance (*g*_*s*_) of treated plants in relation to control plants (plants without stress treatment and application of growth regulators). RTI was obtained using the equation adapted from Chávez-Arias et al. (2020).

All physiological variables described above were determined and recorded at 55 DAE in each experiment using fully expanded leaves collected from the upper portion of the canopy. Also, the measurements were carried out inside the growth chamber to avoid altering the environmental conditions in which the plants were grown.

### Experimental Design and Data Analysis

Data from the first and second experiments were analyzed together as a series of experiments. Each treatment group consisted of five plants, and each plant was an experimental unit. An analysis of variance (ANOVA) was performed (*P* ≤ 0.05). When significant differences were found, the comparative Tukey *post hoc* test was used at *P* ≤ 0.05. Percentage values were transformed using the arcsine function. Data were analyzed using the Statistix v 9.0 software (Analytical Software, Tallahassee, FL, USA), and SigmaPlot (version 10.0; Systat Software, San Jose, CA, USA) was used for the figures. A principal analysis of components was performed with InfoStat 2016 software (analytical software, Universidad Nacional de Córdoba, Argentina) to identify the better plant growth regulators in the study.

## Results

Table 1 summarizes the analysis of variance that shows the effect of the experiments, the different treatments, and their interaction on leaf photosynthetic pigments (chlorophyll *a, b*, total content, and carotenoids), malondialdehyde (MDA) and proline contents, stomatal conductance (*g*_*s*_), relative water content (RWC), chlorophyll content, chlorophyll α fluorescence parameters, plant canopy temperature (PCT) (°C), crop stress index (CSI), and relative tolerance index of rice plants at 55 DAE.

**Table 1:** Summary of the analysis of variance between experiment (genotypes) and heat stress treatments on the physiological and biochemical variables of rice plants.

**Source of variation**	**df**	***g_***s***_***	**RWC**	**CC**	**F_**v**_/F_**m**_**	**F_**0**_**	**F_**m**_**	**F_**v**_**	**F_**v**_/F_**0**_**	**F_**m**_/F_**0**_**	**PCT (^**°**^C)**	**CSI**	**RTI**	**Chl *a***	**Chl *b***	**Chl total**	**Cx+c**	**MDA**	**Proline**
Experiment (*E*)	1	<0.001	0.0183	<0.001	<0.001	<0.001	0.728	<0.001	<0.001	<0.001	<0.05	0.767	<0.001	<0.001	0.107	<0.001	<0.001	0.484	<0.001
Treatment (*T*)	5	<0.001	<0.001	<0.001	<0.001	0.0542	<0.001	<0.001	<0.001	<0.001	<0.001	<0.001	<0.001	<0.001	<0.001	<0.001	<0.001	<0.001	<0.001
*E* x *T*	5	<0.001	<0.05	<0.001	<0.001	0.361	0.280	<0.05	0.296	0.312	<0.05	0.679	<0.001	<0.001	<0.001	<0.001	<0.001	<0.001	<0.001
C.V. (%)		7.58	3.05	5.16	3.93	15.26	5.31	2.27	9.64	8.20	1.61	12.12	8.81	6.92	8.67	16.50	8.97	54.15	32.77

*g_s_, stomatal conductance; RWC, relative water content; CC, chlorophyll content; F_v_/F_m_, maximum quantum efficiency of PSII; initial fluorescence (F0), maximum fluorescence (Fm), variable fluorescence (Fv), maximum efficiency of PSII (Fv/Fm), maximum quantum yield of PSII photochemistry (Fv/F0), and the Fm/F0 ratio, PCT, plant canopy temperature (°C), decrease in the maximum quantum efficiency of PSII; CSI, crop stress index; RTI, relative tolerance index; Chl a, chlorophyll a; Chl b, chlorophyll b; Chl total, total chlorophyll; Cx+c, carotenoids; MDA, Malondialdehyde. C.V., coefficient of variation*.

### Leaf Photosynthetic Pigments, Relative Chlorophyll Content, and Chlorophyll α Fluorescence Parameters

Differences (*P* ≤ 0.01) in the interaction between experiments and treatments on leaf photosynthetic pigments, relative chlorophyll content (Atleaf readings), and the chlorophyll α fluorescence parameters are shown in Table 2. High day/nighttime temperatures improved the total chlorophyll and carotenoids contents. Rice seedlings without any foliar plant hormone sprays (2.36 mg g^−1^ for “F67,” and 2.56 mg g^−1^ for “F2000”) showed a lower total chlorophyll content compared to plants grown at optimal temperature conditions (2.67 mg g^−1^ for “F67,” and 2.80 mg g^−1^ for “F2000”) in both experiments. Additionally, rice seedlings under heat stress and treated with AUX and GA sprays also showed a decrease in the chlorophyll content in both genotypes (AUX = 1.96 mg g^−1^ and GA = 1.45 mg g^−1^ for “F67;” AUX = 2.24 mg g^−1^ and GA = 1.43 mg g^−1^ for “F2000”) under heat stress conditions. Foliar BR treatments caused a slight increase in this variable in both genotypes under heat stress conditions. Finally, foliar CK sprays showed the highest photosynthetic pigment values of all treatments (AUX, GA, BR, SC, and AC treatments) in genotypes F67 (3.24 mg g^−1^) and F2000 (3.65 mg g^−1^). The relative chlorophyll content (Atleaf units) was also diminished by the combined heat stress. The highest values were also recorded in plants sprayed with CK in both genotypes (41.66 for “F67”, and 49.30 for “F2000”). The F_v_ and F_v_/F_m_ ratio showed significant differences between the treatments and cultivars (Table 2). In general, the cultivar F67 was less affected by heat stress than “F2000” in these variables. The F_v_ and F_v_/F_m_ ratio were more affected in the second experiment. Stressed “F2000” seedlings without any plant hormone sprays registered the lowest F_v_ (2120.15) and F_v_/F_m_ ratio (0.59) values; however, foliar CK sprays helped these variables to recover (F_v_: 2591.89, and F_v_/F_m_ ratio: 0.73), obtaining similar readings to those recorded in “F2000” plants grown under optimal temperature conditions (F_v_: 2955.35, and F_v_/F_m_ ratio: 0.72). There were no significant differences in the initial fluorescence (F_0_), maximum fluorescence (F_m_), maximum quantum yield of PSII photochemistry (F_v_/F_0_), and the F_m_/F_0_ ratio. Finally, BR showed similar trends to those observed with the application of CK (2545.06 for F_v_ and 0.73 for F_v_/F_m_ ratio).

**Table 2:** Effect of combined heat stress (40°/30°C day/night) on leaf photosynthetic pigments [total chlorophyll (Chl total), chlorophyll *a* (Chl *a*), chlorophyll *b* (Chl *b*) and carotenoids Cx+c)], relative chlorophyll content (Atleaf units), and chlorophyll fluorescence parameters (initial fluorescence (F_0_), maximum fluorescence (F_m_), variable fluorescence (F_v_), maximum efficiency of PSII (F_v_/F_m_), maximum quantum yield of PSII photochemistry (F_v_/F_0_), and the F_m_/F_0_ ratio) in plants of two rice genotypes [Fedearroz 67 (F67) and Fedearroz 2000 (F2000)] at 55 days after emergence (DAE).

		**Chlorophyll pigments**					**Chlorophyll fluorescence parameters**					
**Genotype**	**Treatment**	**Chl totalmg g^**−1**^ (FW)**	**Chl *a* mg g^**−1**^ (FW)**	**Chl *b*mg g^**−1**^ (FW)**	**Cx+cmg g^**−1**^ (FW)**	**Atleaf units**	**F_**0**_**	**F_**m**_**	**F_**v**_**	**F_**v**_/F_**m**_**	**F_**v**_/F_**0**_**	**F_**m**_/F_**0**_**
F67	AC	2.67cd	2.08 cd	0.59 cd	0.45 cd	41.93 bc	829.75	3863.75	3029.98 a	0.78 ab	3.66	4.66
(Experiment 1) 1)	SC	2.36 def	1.79 de	0.56 d	0.52 bc	33.50 d	814.50	3278.00	2456.79 cd	0.75 bcd	3.02	4.03
	AUX	1.95f	1.36 fg	0.59 cd	0.14 g	38.0 cd	733.50	3352.75	2605.44 bc	0.78 abc	3.56	4.58
	GA	1.45g	1.07 g	0.38 ef	0.27 f	37.59 cd	814.00	3327.25	2513.07 bc	0.76 abcd	3.16	4.16
	CK	3.25 ab	2.52 b	0.73 ab	0.59 ab	41.66 bc	751.00	3579.25	2826.34 ab	0.79 ab	3.76	4.76
	BR	2.14 ef	1.65 ef	0.49 de	0.38 de	40.30 bc	763.75	3469.50	2745.60 abc	0.79 a	3.61	4.56
F2000	AC	2.80 c	2.19 c	0.60 bcd	0.53 bc	48.92 a	1061.40	4016.75	2955.35 a	0.74 cde	2.79	3.79
(Experiment 2)	SC	2.56 cde	2.09 cd	0.54 d	0.50 bc	33.72 d	1266.45	3218.25	2120.15 de	0.66fg	1.76	2.67
	AUX	2.24 ef	1.70 e	0.54 d	0.35 ef	47.45 a	1041.47	3462.25	2420.78 cd	0.70 ef	2.34	3.34
	GA	1.43 g	1.09 g	0.33 f	0.26 f	39.07 c	1112.23	3063.50	1951.27 e	0.64 g	1.76	2.76
	CK	3.65 a	2.87 a	0.78 a	0.66 a	49.30 a	918.86	3510.75	2591.89 bc	0.74 cde	2.82	3.82
	BR	2.88 bc	2.19c	0.69 abc	0.48 cd	44.52 ab	940.69	3485.75	2545.06 bc	0.73 de	2.72	3.72
Significance		***	***	***	***	***	NS	NS	*	**	NS	NS

The treatments evaluated in each genotype were: absolute control (AC), heat stress control (SC), heat stress + auxins (AUX), heat stress + gibberellins (GA), heat stress + cytokinins (CK), and heat stress + brassinosteroids (BR). Data represents the mean of five data ± standard error (n = 5). Data followed by different letters indicate statistically significant differences according to the Tukey test (P ≤ 0.05). Equal letters indicate that means are not statistically significant ( ≤ 0.05). NS, *, ** or *** not significant or significant at P ≤ 0.05, 0.01 or 0.001, respectively.

### Relative Water Content (RWC), Stomatal Conductance, MDA Production, and Proline Content

**Figure 1:**
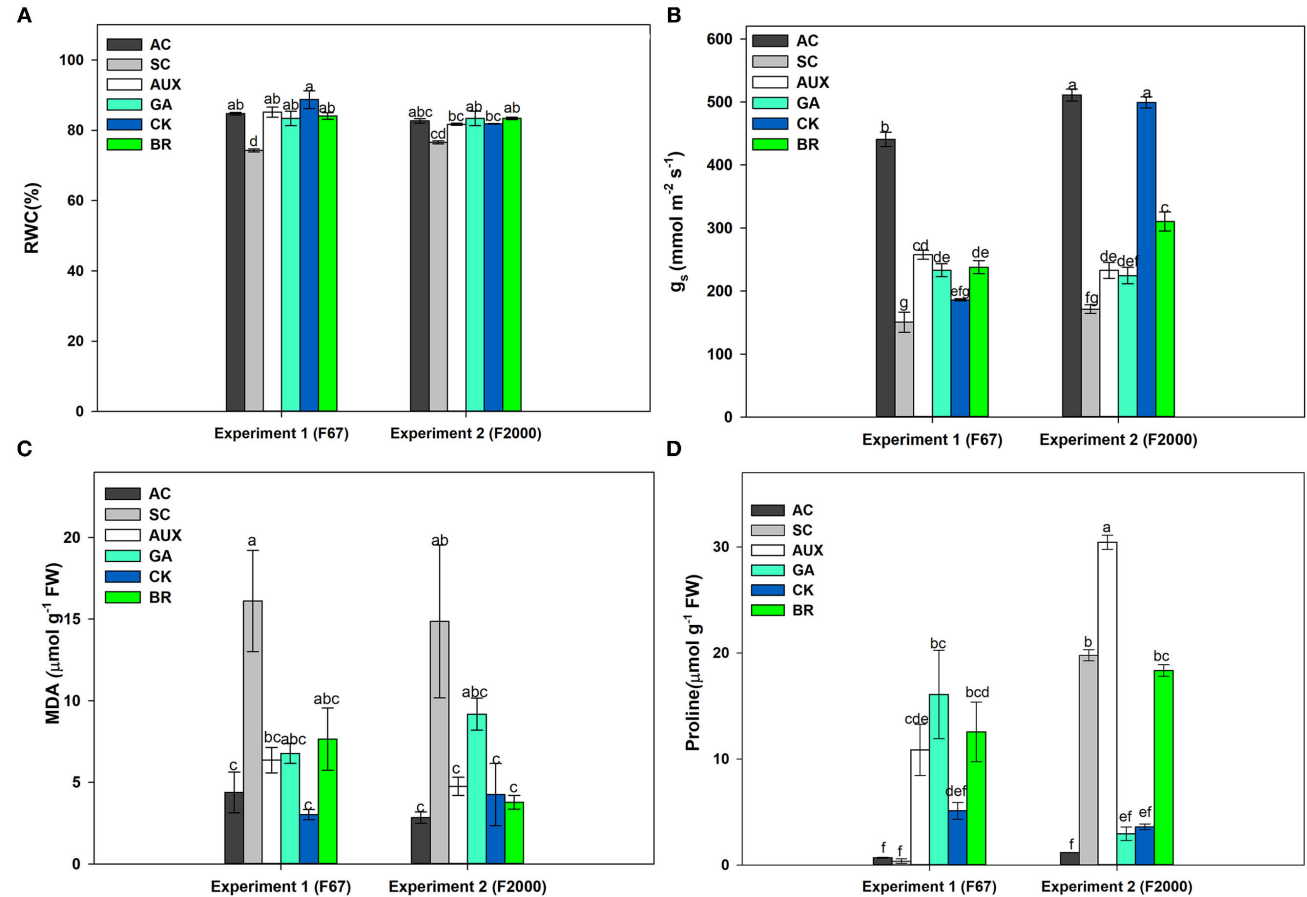
Effect of combined heat stress (40°/30°C day/night) on the relative water content (RWC) **(A)**, stomatal conductance (*g*_*s*_) **(B)**, malondialdehyde production (MDA) **(C)**, and proline content **(D)** in plants of two rice genotypes (F67 and F2000) at 55 days after emergence (DAE). The treatments evaluated in each genotype were: absolute control (AC), heat stress control (SC), heat stress + auxins (AUX), heat stress + gibberellins (GA), heat stress + cytokinins (CK), and heat stress + brassinosteroids (BR). Each column represents the mean of five data ± standard error (*n* = 5). Bars followed by different letters indicate statistically significant differences according to the Tukey test (*P* ≤ 0.05). Equal letters indicate that means are not statistically significant ( ≤ 0.05).

The relative water content (RWC) of rice plants subjected to the different treatments showed differences (*P* ≤ 0.05) in the interaction between experiments and foliar treatments (Figure 1A). The SC treatment registered the lowest values in both genotypes (74.01% for F67 and 76.6% for F2000). A significant increase in RWC was registered in rice plants of both genotypes treated with different plant hormones under heat stress. In general, the foliar CK, GA, AUX or BR applications enhanced RWC, reaching similar values to those of plants grown under optimal conditions during the experiments. The values recorded for the absolute control and foliar sprayed plants were around 83% for both genotypes. On other hand, *g*_*s*_ also showed significant differences (*P* ≤ 0.01) in the interaction between experiments and treatments (Figure 1B). Absolute control plants (AC) also registered the highest values in each genotype (440.65 mmol m^−2^s^−1^ for F67 and 511.02 mmol m^−2^s^−1^ for F2000). Rice plants only subjected to combined heat stress showed the lowest *g*_*s*_ values for both genotypes (150.60 mmol m^−2^s^−1^ for F67 and 171.32 mmol m^−2^s^−1^ for F2000). *g*_*s*_ was also enhanced by foliar applications of all plant growth regulators. The effects of foliar plant hormone sprays were more notorious in “F2000” rice plants sprayed with CK. This group of plants did not show differences compared to the absolute control plants (511.02 for AC and 499.25 mmol m^−2^s^−1^for CK).

MDA (*P* ≤ 0.01) and proline (*P* ≤ 0.01) contents also showed significant differences in the interaction between experiments and plant hormone treatments (Figures 1C,D). An increase was observed in lipid peroxidation in SC treatments in both genotypes (Figure 1C); however, plants treated with foliar growth regulator sprays showed a decrease in lipid peroxidation for both genotypes. In general, the use of plant hormones (CK, AUX, BR, or GA) caused a reduction in lipid peroxidation (MDA content). Differences were not obtained between AC plants and plants under heat stress and sprayed with plant hormones in both genotypes (observed values between 4.38 and 6.77 μmol g^−1^ of FW in “F67” plants and 2.84 and 9.18 μmol g^−1^ of FW “F2000” plants). On the other hand, proline synthesis was lower in “F67” plants than in “F2000” plants under combined stress. The foliar AUX or BR applications also caused a higher proline production in rice plants subjected to heat stress in both experiments. It was observed that the use of these hormones notoriously increased the amino acid content (30.44 and 18.34 μmol g^−1^ of FW for AUX and BR, respectively) in “F2000” plants (Figure 1D).

### Plant Canopy Temperature, Relative Tolerance Index (RTI), and Crop Stress Index (CSI)

**Figure 2:**
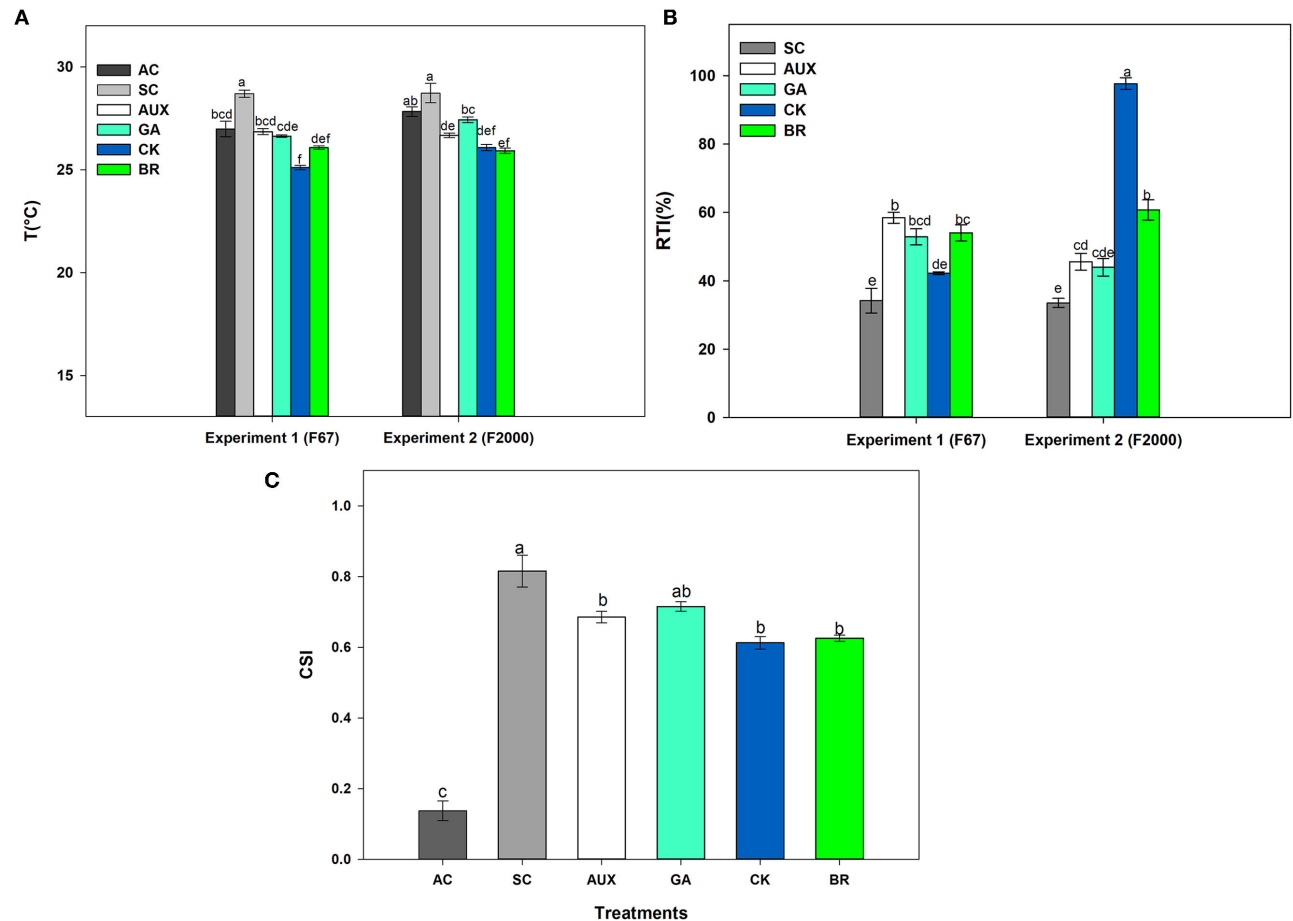
Effect of combined heat stress (40°/30°C day/night) on plant canopy temperature **(A)**, the relative tolerance index (RTI) **(B)**, and crop stress index (CSI) **(C)** of two commercial rice genotypes (F67 and F2000) exposed to different heat treatments. The treatments evaluated in each genotype were: absolute control (AC), heat stress control (SC), heat stress + auxins (AUX), heat stress + gibberellins (GA), heat stress + cytokinins (CK), and heat stress + brassinosteroids (BR). The combined heat stress consisted of exposing rice plants to high day/nighttime temperatures (40°/30°C day/night). Each column represents the mean of five data ± standard error (*n* = 5). Bars followed by different letters indicate statistically significant differences according to the Tukey test (*P* ≤ 0.05). Equal letters indicate that means are not statistically significant ( ≤ 0.05).

The effect of foliar plant growth regulator sprays and combined heat stress on plant canopy temperature, and relative tolerance index (RTI) is shown in Figures 2A,B. AC plants registered temperatures close to 27°C on the plant canopy whereas SC plants showed a canopy temperature around 28°C for both genotypes. It was also observed that foliar CK and BR treatments caused a higher decrease in the plant canopy temperature between 2 and 3°C compared to SC plants (Figure 2A). The RTI showed similar behavior to the other physiological variables, showing significant differences (*P* ≤ 0.01) in the interactions between experiments and treatments (Figure 2B). SC plants showed lower plant tolerance in both genotypes (34.18 and 33.52% for “F67” and “F2000” rice plants, respectively). The foliar application of plant hormones improved the RTI in plants exposed to stressful temperatures. This effect was more notorious in “F2000” plants sprayed with CK, registering an RTI of 97.69. On the other hand, significant differences were only observed on the crop stress index (CSI) of rice plants subjected to the stress condition (*P* ≤ 0.01) in the factor foliar sprays (Figure 2C). Rice plants only exposed to combined heat stress showed the highest stress index values (0.816). The stress index was lower (values between 0.6 and 0.67) when rice plants were sprayed with the different plant hormones. Finally, rice plants grown under optimal conditions showed a value of 0.138.

### Biplot Analysis of Physiological and Biochemical Responses to Combined Heat Stress Management With Growth Regulators

**Figure 3:**
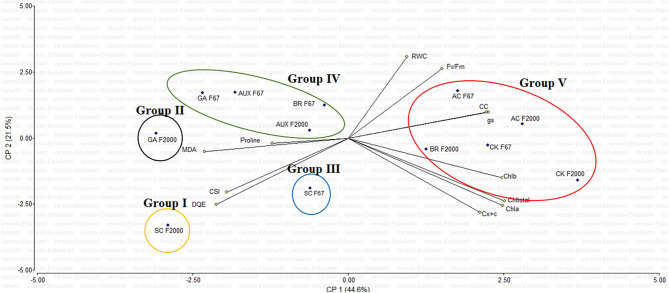
Effect of combined heat stress (40°/30°C day/night) on the biplot analysis of plants of two rice genotypes (F67 and F2000) at 55 days after emergence (DAE). Abbreviations: AC F67, absolute control F67; SC F67, heat stress control F67; AUX F67, heat stress + auxins F67; GA F67, heat stress + gibberellins F67; CK F67, heat stress + cytokinins F67; BR F67, heat stress + brassinosteroids F67; AC F2000, absolute control F2000; SC F2000, heat stress control F2000; AUX F2000, heat stress + auxins F2000; GA F2000, heat stress + gibberellins F2000; CK F2000, heat stress + cytokinins, and BR F2000, heat stress + brassinosteroids F2000.

The principal component analysis (PCA) showed that the variables evaluated at 55 DAE explained 66.1% of the physiological and biochemical responses of heat-stressed rice plants and treated with growth regulator sprays (Figure 3). Vectors represent the variables, whereas points identify plant growth regulators (GR). The vectors of *g*_*s*_, chlorophyll content, maximum quantum efficiency of PSII (F_v_/F_m_), and biochemical readings (TChl, MDA, and proline) have angles close to the origin, showing that there is a high correlation between plant physiological behavior and these variables. The rice seedlings grown at optimal temperatures (AC) and F2000 plants treated with CK and BR form a single group (V). Also, most of the plants treated with GR form another single group (IV), and the GA treatment in F2000 forms a single group (II). In contrast, heat-stressed rice seedlings without any foliar plant hormone sprays (SC for both genotypes) (group I and III) are located in the sector opposite to group V, showing a negative effect of heat stress on plant physiological and biochemical responses.

## Discussion

Variables such as chlorophyll content, stomatal conductance, F_v_/F_m_ ratio, CSI, MDA, RTI, and proline content are useful to understand the rice genotypes acclimatization and evaluate the impact of agronomic strategies under heat stress (Sarsu et al., 2018; Quintero-Calderón et al., 2021). The objective of the experiment was to evaluate the effect of the application of four growth regulators on the physiological and biochemical variables of rice seedlings subjected to combined heat stress. The seedling test is a simple and rapid method that allows the simultaneous evaluation of rice plants depending on the size or conditions of the available infrastructure (Sarsu et al., 2018). The results of this study showed that the combined heat stress caused different physiological and biochemical responses on both rice genotypes, indicating an acclimatization process. These results also showed that the foliar growth regulator sprays (principally cytokinins and brassinosteroids) helped rice acclimatization to combined heat stress since *g*_*s*_, RWC, and F_v_/F_m_ ratio, photosynthetic pigments, and proline content were mainly favored.

The application of growth regulators helped the plant water status of rice plants under heat stress conditions, which can be associated with a higher *g*_*s*_ and lower plant canopy temperature. This study showed that rice plants treated mainly with CK or BR registered higher *g*_*s*_ values and lower PCT than SC treatment in “F2000” plants (susceptible genotype). Previous studies have also found that *g*_*s*_ and PCT are accurate physiological indicators to determine the acclimatization response of rice plants and the effect of agronomic strategies to heat stress (Restrepo-Diaz and Garces-Varon, 2013; Sarsu et al., 2018; Quintero-Calderón et al., 2021). Foliar CK or BR enhanced *g*_*s*_ under stress conditions since these plant hormones can help the stomatal opening by interacting with the synthesis of other signaling molecules such as ABA (stomatal closing promoter under abiotic stress) (Macková et al., 2013; Zhou et al., 2014). Stomatal opening promotes leaf cooling and helps to decrease canopy temperature (Sonjaroon et al., 2018; Quintero-Calderón et al., 2021). For these reasons, plant canopy temperature could be lower in rice plants sprayed with CK or BR under combined heat stress.

The high-temperature stresses diminish the content of photosynthetic pigments in leaves (Chen et al., 2017; Ahammed et al., 2018). In this study, the photosynthetic pigments tended to decrease in both genotypes when rice plants were under heat stress conditions and without any plant growth regulator sprays (Table 2). Feng et al. (2013) also reported a substantial decrease in the leaf chlorophyll content in two wheat genotypes exposed to heat stress. Exposure to high temperatures usually results in low chlorophyll content that can be caused by decreased chlorophyll biosynthesis, pigment degradation, or their combined effect under heat stress (Fahad et al., 2017). However, rice plants mainly treated with CK and BR increased the leaf photosynthetic pigment concentration under heat stress. Similar results were found by Jespersen and Huang (2015) and Thussagunpanit et al. (2015) who observed that leaf chlorophyll values increased after the application of Zeatin and epibrassinolide hormones in creeping bentgrass and rice plants exposed to heat stress, respectively. A plausible explanation why CK and BR helped the leaf chlorophyll content under combined heat stress is because CK can up-regulate the initiation of continuous induction of expression promoters (such as senescence-activated promoter (SAG12) or HSP18 promoter), reducing leaf chlorophyll loss, delaying leaf senescence, and enhancing plant heat resistance (Liu et al., 2020). BR can protect leaf chlorophyll and improve leaf chlorophyll content by activating or inducing the synthesis of enzymes involved in chlorophyll biosynthesis under stress (Sharma et al., 2017; Siddiqui et al., 2018). Finally, both plant hormones (CK and BR) also help the expression of heat shock proteins, improving different metabolic acclimatization processes such as the increase in chlorophyll biosynthesis (Sharma et al., 2017; Liu et al., 2020).

The parameters of chlorophyll *a* fluorescence are a fast and non-destructive technique that allows estimating the plant's tolerance or acclimatization to abiotic stress conditions (Chaerle et al., 2007; Kalaji et al., 2017). Parameters such as the F_v_/F_m_ ratio have been used as plant acclimatization indicators to stressful conditions (Alvarado-Sanabria et al., 2017; Chávez-Arias et al., 2020). In this research, SC plants showed the lowest values of this variable, mainly in “F2000” rice plants. Yin et al. (2010) also found that the F_v_/F_m_ ratio significantly decreased at temperatures higher than 35°C in rice leaves at maximum tillering. According to Feng et al. (2013), a low F_v_/F_m_ ratio under heat stress suggests a decrease in the capture and conversion rate of excitation energy by PSII reaction centers, indicating disorganization of PSII reaction centers under heat stress conditions. This observation allows us to infer that the disorder in the photosynthetic machinery appeared to be more pronounced in sensitive cultivars (Fedearroz 2000) than in resistant ones (Fedearroz 67).

The use of CK or BR mainly enhanced PSII efficiency under combined heat stress. Similar results were obtained by Thussagunpanit et al. (2015), who observed that the application of BR enhanced the PSII efficiency under heat stress in rice plants. Kumar et al. (2020) also found that chickpea plants treated with CK (6-Benzyladenine) and exposed to heat stress increased the F_v_/F_m_ ratio, concluding that foliar CK application helped the activity of PSII by activating the operation of the zeaxanthin pigment cycle. Also, foliar BR sprays favored the PSII photosynthetic machinery under combined stress conditions, suggesting that the application of this plant hormone resulted in lower dissipation of excitation energy in the PSII antennae and promoted the accumulation of chloroplastic small heat shock proteins (Ogweno et al., 2008; Kothari and Lachowiec, 2021).

The MDA and proline content generally increased when plants were under abiotic stress compared to plants grown under optimal conditions (Alvarado-Sanabria et al., 2017). Previous studies also identified that MDA and proline levels are biochemical makers used to understand the acclimatization process or the effect of agronomic practices in rice plants under high day or nighttime temperatures (Alvarado-Sanabria et al., 2017; Quintero-Calderón et al., 2021). Those studies also reported that MDA and proline contents were generally higher in rice plants exposed to high night or daytime temperatures, respectively. However, the application of foliar CK and BR helped to decrease MDA and increase proline levels, mainly in the tolerant genotype (Fedearroz 67). CK sprays could help the overexpression of cytokinin oxidase/dehydrogenase that increased the content of protective compounds such as betaine, proline, etc. (Liu et al., 2020). BR promote the induction of osmoprotectants such as betaines, sugars, and amino acids, including free proline, maintaining cell osmotic balance under many unfavorable environmental conditions (Kothari and Lachowiec, 2021).

The crop stress index (CSI) and relative tolerance index (RTI) are used to determine if the treatments evaluated help to mitigate different stresses (abiotic and biotic) and have a positive effect on plant physiology (Castro-Duque et al., 2020; Chávez-Arias et al., 2020). CSI values can range between zero and one, representing the non-stressed and stressed conditions, respectively (Lee et al., 2010). The values of heat-stressed plants (SC) showed a CSI between 0.8 and 0.9 (Figure 2C), suggesting that rice plants were negatively affected under combined stress. However, foliar BR (0.6) or CK (0.6) sprays mainly caused a decrease in this index under abiotic stress compared to SC rice plants. In “F2000” plants, the RTI showed a higher increase with the use of CK (97.69%), and BR (60.73%) compared to SC (33.52%), suggesting that these plant growth regulators also helped to improve rice tolerance to combined heat stress. These indexes have been suggested for the management of stressful situations in different species. Studies performed by Lee et al. (2010) showed that two cotton cultivars under moderate water stress had a CSI around 0.85, whereas well-irrigated cultivars registered CSI values between 0.4 and 0.6, concluding that this index is an indicator of the cultivar adaptability to water stress conditions. Additionally, Chávez-Arias et al. (2020) evaluated the effect of synthetic elicitors as a strategy to mitigate combined stress in cape gooseberry plants, finding that plants sprayed with these compounds displayed a higher RTI (65%) compared to plants only exposed to combined stress (biotic and abiotic stress). Based on the above, CK and BR can be considered an agronomic strategy to induce rice tolerance to combined heat stress, since these plant growth regulators caused positive biochemical and physiological responses.

**Figure 4:**
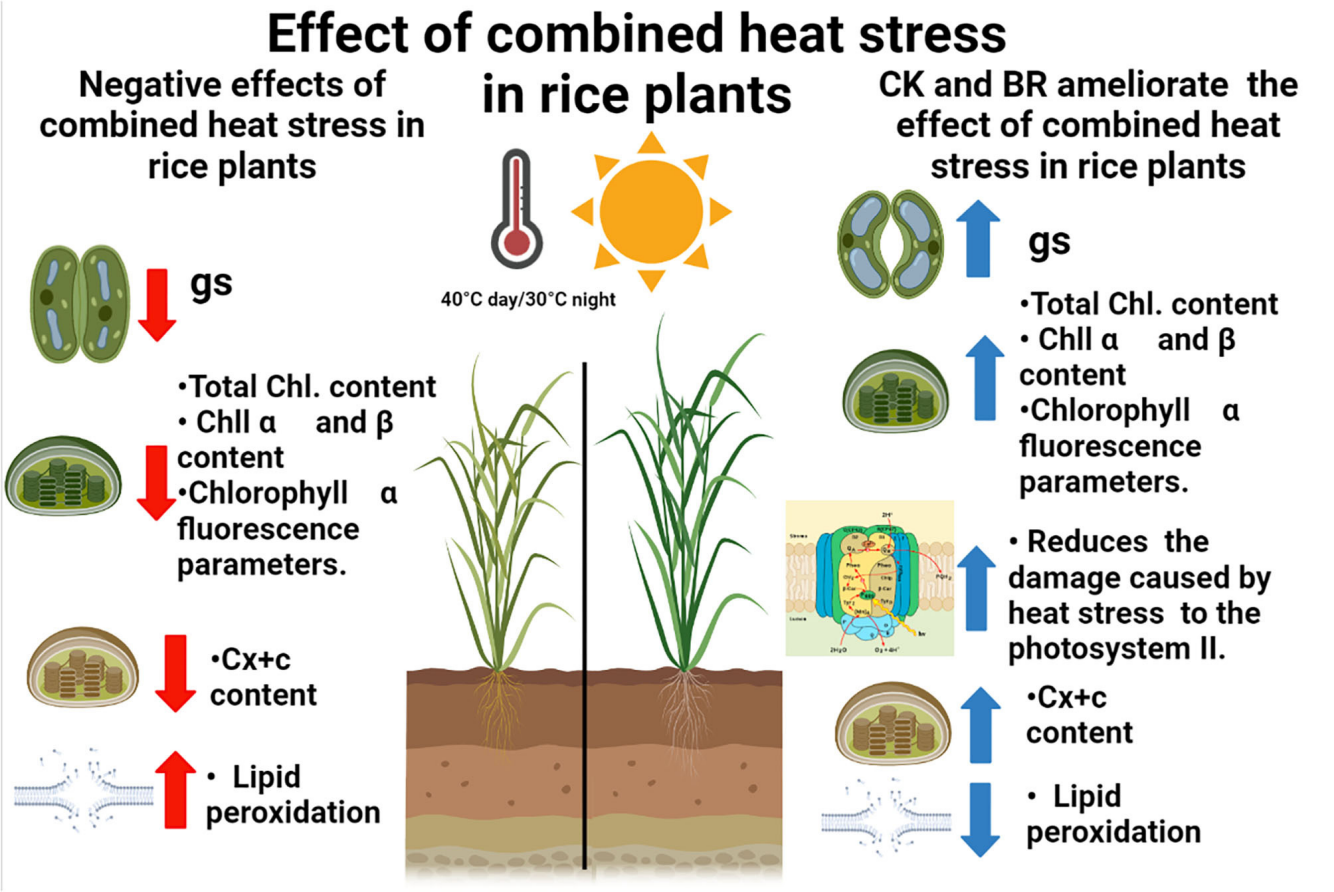
Concept model of the impact of the combined heat stress and foliar plant growth regulators sprays in rice plants. Red arrows and blue arrows indicate the negative or positive effect of interaction between combined heat stress and foliar BR (brassinosteroids) and CK (cytokinins) applications on the physiological and biochemical responses, respectively. gs: stomatal conductance; Total Chl: total chlorophyll content; Chl α: Chlorophyl α content; Chl β: Chlorophyl β content; Cx+c: Carotenoids content.

In the last years, rice studies in Colombia have focused on the evaluation of genotypes with resistance to high day or nighttime temperatures using physiological or biochemical traits (Sánchez-Reinoso et al., 2014; Alvarado-Sanabria et al., 2021). However, the analysis of practical, economic, and profitable techniques has gained importance in the last years in order to propose crop integrated management to ameliorate the effects of combined heat stress periods in the country (Calderón-Páez et al., 2021; Quintero-Calderón et al., 2021). For this reason, the physiological and biochemical responses of rice plants to combined heat stress (40°C day/30°C night) observed in this study indicate that the foliar CK or BR sprays may be an appropriate crop management technique to mitigate the adverse effects of moderate heat stress periods. These treatments increased the tolerance (low CSI and high RTI) of the two rice genotypes, showing common trends in the physiological and biochemical responses of plants under combined heat stress. The principal responses of rice plants were a decrease in *g*_*s*_, total chlorophyll, Chl α and β, and carotenoids content. Additionally, plants registered an increase in the damage of the PSII (reducing the values of chlorophyll fluorescence parameters such as F_v_/F_m_ ratio), and lipid peroxidation. On the other hand, these negative effects were mitigated and the proline content increased when rice plants were treated with CK and BR (Figure 4).

In summary, the physiological and biochemical responses of this study revealed that Fedearroz 2000 rice plants were more susceptible to the combined heat stress period than Fedearroz 67 rice plants. All growth regulators evaluated in this research (auxins, gibberellins, cytokinins, or brassinosteroids) demonstrated a certain level of mitigation of combined heat stress. However, cytokinins and brassinosteroids induced better plant acclimatization, since both plant growth regulators increased the chlorophyll content, chlorophyll α fluorescence parameters, *g*_*s*_, and RWC and decreased MDA content and canopy temperature, compared to rice plants without any application. The foregoing allows us to conclude that the use of plant growth regulators (cytokinins and brassinosteroids) is a useful tool for managing stress conditions due to severe heat stress in rice crops when periods of high temperatures are expected.

## Data Availability Statement

The original contributions presented in the study are included in the article, further inquiries can be directed to the corresponding author.

## Author Contributions

AP-B and HR-D: conceptualization, writing—review, editing, data curation, and formal analysis. AP-B, HR-D, and GG-V: methodology, investigation, writing—original draft, and validation. HR-D and GG-V: resources, supervision, project administration, and funding acquisition. All authors agreed to be accountable for the content of the work.

## Conflict of Interest

The authors declare that the research was conducted in the absence of any commercial or financial relationships that could be construed as a potential conflict of interest.

## Publisher's Note

All claims expressed in this article are solely those of the authors and do not necessarily represent those of their affiliated organizations, or those of the publisher, the editors and the reviewers. Any product that may be evaluated in this article, or claim that may be made by its manufacturer, is not guaranteed or endorsed by the publisher.
