# Supplementary material for: Foliar Growth Regulator Sprays Induced Tolerance to Combined Heat Stress by Enhancing Physiological and Biochemical Responses in Rice
Source: Front Plant Sci. 2021 Jul 23;12:702892. doi: 10.3389/fpls.2021.702892 (PMC8343023; doi:10.3389/fpls.2021.702892)
Supplement: Supplementary file 1 [file Table_1.pdf]

Table supplementary 1. Chemical name of the active ingredient, commercial name, and manufacturer of the growth regulators used to evaluate foliar applications in rice plants under combined heat stress (40°/30°C day/nigttime).

Chemical name of the active ingredient	Commercial name (manufacturer)	Biological activity of the compound
**1-Naphthaleneacetic acid**	HORMONAGRO® A.N.A (Colinagro, Bogotá, Colombia)	It stimulates stomatal opening and the production of small heat shock proteins (sHSPs) (Ha et al., 2012)
**Gibberellic acid**	Gibberellic acid (Dr. Ehrenstorfer GmbH, Augsburg, Germany)	It induces germination and seedling growth and decelerates floral transition (Alonso-Ramirez et al., 2009)
**Trans-Zeatin**	M977 trans-Zeatin, 98% (HPLC) (AK scientific)	It binds to chaperone proteins to mitigate the effect of high temperatures, works as a marker to heat shock protein 90 (HSP90) (Ahammed et al., 2014; Wang et al., 2016)
**Spirostan-6-one, 3,5-dihydroxy-, (3b,5a,25R)**	Biomex DI-31 (Minerales exclusivos SA, Bogotá, Colombia)	It increases the synthesis and accumulation of HSP, photosynthesis, and gas exchange (Dhaubhadel et al., 2002; Ogweno et al., 2007)
